# Impact of autoimmune gastritis on chronic urticaria in paediatric patients – pathophysiological point of views

**DOI:** 10.1007/s00431-023-05324-2

**Published:** 2023-11-10

**Authors:** J. Bufka, J. Sýkora, L. Vaňková, V. Gutová, Š. Kačerová, O. Daum, J. Schwarz

**Affiliations:** 1 Department of Pediatrics, Faculty of Medicine in Pilsen, Faculty Hospital, Charles University in Prague, Alej Svobody 80, Pilsen, 323 00 Czech Republic; 2 Department of Histology and Embryology, Faculty of Medicine in Pilsen, Charles University, Pilsen, Czech Republic; 3 Department of Allergology and Immunology, Teaching Hospital in Pilsen, Pilsen, Czech Republic; 4 Sikl’s Department of Pathology, Faculty of Medicine in Pilsen, Charles University, Pilsen, Czech Republic

**Keywords:** Chronic urticaria, Autoimmune gastritis, Pathophysiology, Paediatrics

## Abstract

We would like to provide an updated comprehensive perspective and identify the components linked to chronic spontaneous urticaria (CSU) without specific triggers in autoimmune atrophic gastritis (AAG). AAG is an organ-specific autoimmune disease that affects the corpus-fundus gastric mucosa. Although we lack a unified explanation of the underlying pathways, when considering all paediatric patients reported in the literature, alterations result in gastric neuroendocrine enterochromaffin-like (ECL) cell proliferation and paracrine release of histamine. Several mechanisms have been proposed for the pathogenesis of CSU, with much evidence pointing towards AAG and ECL cell responses, which may be implicated as potential factors contributing to CSU. The excessive production/release of histamine into the bloodstream could cause or trigger exacerbations of CSU in AAG, independent of *Helicobacter pylori*; thus, the release of histamine from ECL cells may be the primary modulator.

*Conclusion*: Considering the understanding of these interactions, recognising the respective roles of AAG in the pathogenesis of CSU may strongly impact the diagnostic workup and management of unexplained/refractory CSU and may inform future research and interventions in the paediatric population.

**Table:** 

**What is Known:** *• Autoimmune atrophic gastritis is a chronic immune-mediated inflammatory disease characterised by the destruction of the oxyntic mucosa in the gastric body and fundus, mucosal atrophy, and metaplastic changes.* *• Autoimmune atrophic gastritis in paediatric patients is important because of the poor outcome and risk of malignancy and possibly underestimated entities primarily reported in single-case reports.*	
**What is New:** *• Upper gastrointestinal inflammatory disorders, independent of H. pylori, have been implicated as potential inducing factors in the development of chronic spontaneous urticaria.* *• If a paediatric patient presents with symptoms such as anaemia, reduced vitamin B12 levels, recurrent urticaria with no other detectable aetiology, positive anti-parietal cell antibodies, and elevated gastrin levels, autoimmune atrophic gastritis should be considered a possible cause of chronic urticaria.*

**What is Known:**

*• Autoimmune atrophic gastritis is a chronic immune-mediated inflammatory disease characterised by the destruction of the oxyntic mucosa in the gastric body and fundus, mucosal atrophy, and metaplastic changes.*

*• Autoimmune atrophic gastritis in paediatric patients is important because of the poor outcome and risk of malignancy and possibly underestimated entities primarily reported in single-case reports.*

**What is New:**

*• Upper gastrointestinal inflammatory disorders, independent of H. pylori, have been implicated as potential inducing factors in the development of chronic spontaneous urticaria.*

*• If a paediatric patient presents with symptoms such as anaemia, reduced vitamin B12 levels, recurrent urticaria with no other detectable aetiology, positive anti-parietal cell antibodies, and elevated gastrin levels, autoimmune atrophic gastritis should be considered a possible cause of chronic urticaria.*

## Introduction

Urticaria is widely considered a heterogeneous group of diseases that share a distinct skin reaction pattern: the development of urticarial skin lesions. Chronic spontaneous urticaria (CSU) is characterised by episodic itchy wheals that occur on most days of the week for more than six weeks without an evident triggering stimulus within 24 h [1]. The pathogenesis of this condition is complex. Despite careful clinical and laboratory investigations, a clear cause of CSU cannot be identified in approximately 75% of patients, and treatment is ineffective.

CSU can be caused by autoreactivity/autoimmunity in at least one-third of patients [2] and is more often associated with autoimmune diseases (e.g. thyreopathies, celiac disease, rheumatoid arthritis, systemic lupus erythematosus, Sjögren's syndrome, and type 1 diabetes mellitus) [3]. Several studies have raised concerns regarding the possible connection between chronic urticaria and infection, where an autoreactive immune response may be activated in predisposed patients. Infections of the gastrointestinal tract, such as *Helicobacter pylori* (*H. pylori*) infections, viral hepatitis, and parasitosis, may be associated with CSU [4]. The association of chronic urticaria with haematological malignancies has rarely been described. Furthermore, chronic inducible urticaria is less common, and its manifestation depends on the presence of specific triggers, such as physical stimuli, stress, food antigens, and drugs; however, both forms can occur simultaneously.

Autoimmune atrophic gastritis (AAG) is a well-established gastric pathological condition in adults; however, it is rarely described in paediatric patients. AAG is a chronic immune-mediated inflammatory disease characterised by the destruction of the oxyntic mucosa in the gastric body and fundus, mucosal atrophy, metaplastic changes, and the presence of two types of circulating autoantibodies: anti-parietal cell (APCA) and anti-intrinsic factor (AIFA) antibodies [5]. The parietal cells are destroyed by an immune-mediated process leading to hypo- or achlorhydria, which stimulates gastrin-producing G cells in the anthroduodenal region, leading to abnormally elevated gastrin levels, which stimulate gastric proliferation of enterochromaffin-like (ECL) cells. Long-term (and in the absence of parietal cells, ineffective) stimulation of ECL cells leads to hyperplasia, possibly even their transformation into neuroendocrine tumours (NETs) [6]. In advanced stages, when ECL cells become hyperplastic, histamine leaks into the bloodstream. Excess histamine in patients with AAG could trigger the emergence of chronic urticaria, contributing to its further progress.

The connection between gastrointestinal diseases and CSU has long been intensively investigated; however, our knowledge regarding the potential AAG pathogenetic mechanisms and the exact players that trigger CSU remains elusive; therefore, proactive case-finding strategies are needed. In this review, we focus on new pathophysiological aspects of AAG, including hyperplastic ECL cells producing histamine, as potential triggers for CSU. Therefore, close attention should be paid to the occurrence of AAG in children with a history of CSU.

## Definition and classification of AAG in the context of CSU

Upper gastrointestinal (GI) inflammatory disorders, independent of *H. pylori*, have been implicated as potential inducing factors in the development of CSU*.* AAG in paediatric patients is important because of the poor outcome and risk of malignancy and possibly underestimated entities primarily reported in single-case reports. AAG is defined as the loss of appropriate gastric glands with or without metaplasia in the setting of chronic inflammation directed against the structures of gastric epithelial cells, which, in some cases, may be triggered by *H. pylori*. Regardless of the aetiology, the diagnosis of atrophic gastritis should be confirmed by histopathology. Although it has been described for several years, its actual pathophysiological mechanisms, natural history, and possible neoplastic complications are unclear [7]. AAG is characterised by the disruption of gastric epithelial cell development, targeting gastric parietal cells, leading to oxyntic-restricted mucosal atrophy, loss of intrinsic factors, and reduced acid output [5]. AAG is strongly associated with anaemia, atrophy, intestinal metaplasia, and ECL hyperplasia of the gastric fundus and body. These are hallmarks of this condition during childhood [8, 9]. However, to date, the long-term outcomes of AAG, including improvements in gastric atrophy and ECL hyperplasia in children, consistent with the known progression of adult AAG, remain debatable.

AAG is classified into two types according to the underlying etiopathogenic mechanisms: a predominant type in patients with the infection of *H. pylori* or due to other mechanisms of autoimmunity development, mainly limited to the corpus and fundus-dominant advanced atrophy.

The interaction between genetic and environmental factors has been implicated and may often be associated with other autoimmune diseases. In juvenile populations, autoimmune thyroid disease may be associated [10].

*H. pylori* infection may trigger an immune reaction against the antigens of the oxyntic mucosa itself, either based on cross-reactivity with *H. pylori* antigens or as a result of the stimulation of the immune system by neoantigens arising during tissue damage by inflammation. Several important factors help differentiate AAG from *H. pylori*-induced gastritis, including ECL hyperplasia, parietal cell pseudohypertrophy, and oxyntic gland involvement [11].

Identifying prolonged upper gastric inflammatory disorders that may lead to CSU and trigger exacerbations independent of *H. pylori* infection is important [12]. Hence, this might imply that in patients positive for *H. pylori*, urticaria is not linked to the bacterium itself or immunological reactions against the bacterium, but rather to the underlying inflammation [12]. Furthermore, in accordance with these findings, it was recently demonstrated that *H. pylori*-negative peptic ulcer disease and gastritis are comorbidities associated with an increased risk of CSU [12, 13]. Thus, as far as CSU alterations are concerned, these exciting findings suggest the importance of gastroenterological workup in paediatric patients with CSU independent of *H. pylori.* However, our understanding of the mechanisms, context, and roles of inflammation, ECL hyperplasia responses, and pathology in CSU is constantly evolving.

## Discussed pathophysiological mechanism of chronic urticaria in AAG

To elucidate the complexity of the pathophysiology of AAG and its impact on the possible development of CSU, briefly discussing the histology and cellular composition of gastric glands, as well as their connections to various physiological processes and conditions would be useful.

The simple columnar mucus-secreting epithelium covers the entire luminal surface and comprises a continuous layer of surface mucous cells that release gastric mucus from the apical surface to form a thick protective layer over the gastric lining. Although all the gastric glands are tubular, they vary in form and cellular composition in different parts of the stomach. They can be divided into three groups as follows: the cardiac glands (glandulae cardiacae), principal glands in the body and fundus (glandulae gastricae propriae), and pyloric glands (glandulae pyloricae) [14]. Mucus-secreting cells are predominant in the cardiac glands. The other cell types were either absent or very few.

The pyloric glands are located in the pyloric antrum. These cells are primarily mucus-secreting cells. Parietal and chief cells are rare. By contrast, neuroendocrine cells are numerous, especially G cells, which secrete gastrin.

**Fig. 1:**
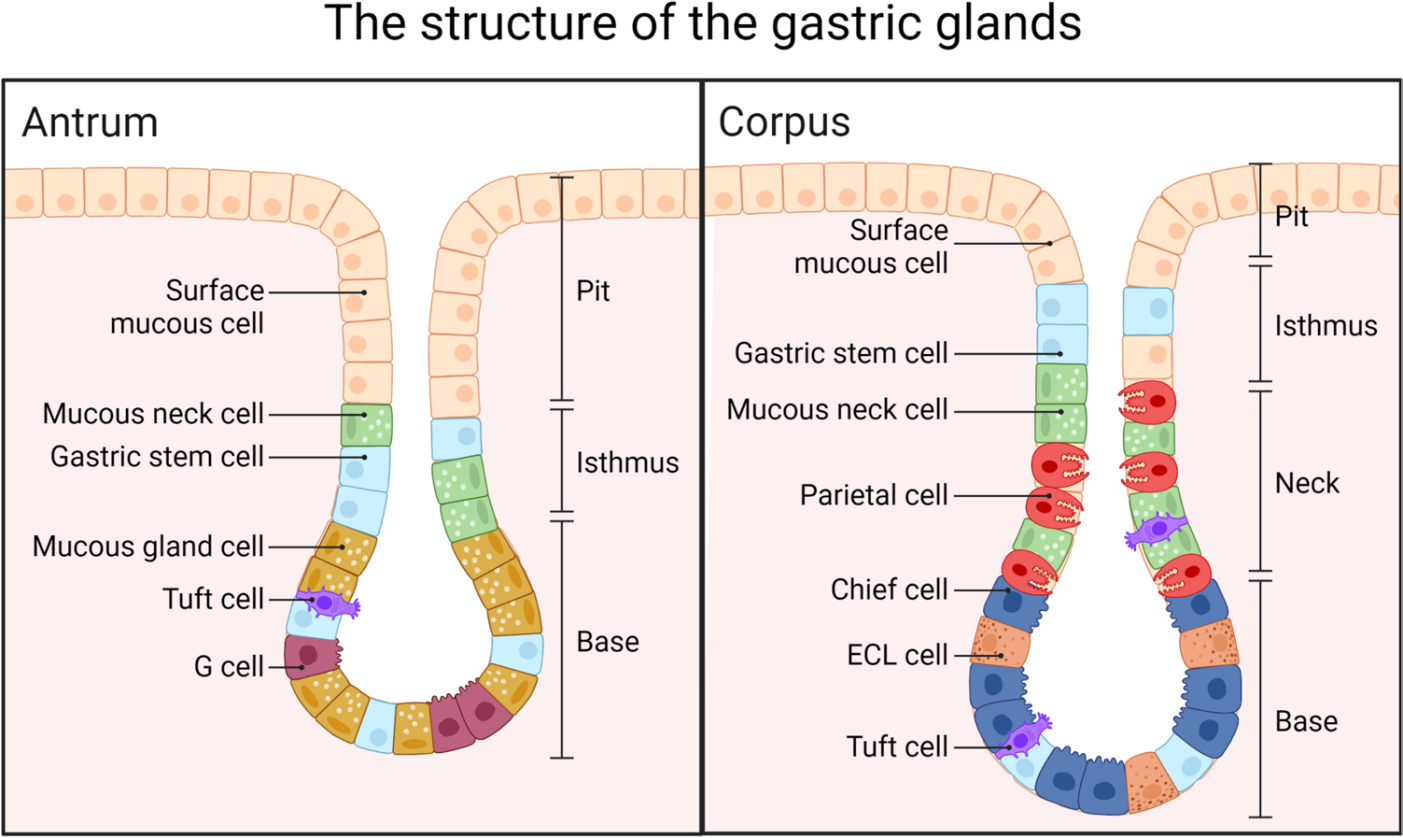
Structure of the gastric glands. Adapted from “Cells of Gastric Glands (Antrum vs Corpus)”, by BioRender.com (2023). Retrieved from https://app.biorender.com/biorender-templates

At least five distinct cell types are present in the walls of the principal glands: chief, parietal, mucous neck, stem, and neuroendocrine. The chief (zymogenic) cells are predominant in the lower regions of the gastric glands. They are strongly basophilic (owing to their secretory function) and contain numerous granules with the inactive enzyme pepsinogen, a precursor of pepsin. Parietal (oxyntic) cells are present in the mucous neck and throughout the deeper parts of the glands. They are a source of hydrogen ions (which create hydrochloric acid in the lumen) and an intrinsic glycoprotein necessary for the absorption of vitamin B_12_. Mucous neck cells are mainly localised in the necks of the gastric glands. These cells include many progenitors and immature surface mucous cells. Their primary function is to secrete mucus. Stem cells are predominantly located in the isthmus (the border between the gastric pits and gastric glands). These cells undergo mitosis, migration, and differentiation into various types of gastric cells. Among the chief cells, the neuroendocrine (enteroendocrine) cells are situated mainly in the deeper parts of the glands. These cells synthesise several biogenic amines and polypeptides that are important for controlling motility and glandular secretion. In the stomach, these include G cells that secrete gastrin, D cells that produce somatostatin, and ECL cells (Fig. 1) [15].

In addition to these basic cell types, other rarer and less-explored populations of cells are present in the gastric glands, such as tuft cells. Tuft cells are characterised by the presence of a luminally directed tuft that displays a distinct membrane-covered array of microtubules. The presence of the apical tuft apparatus suggests that tuft cells function in the detection and transmission of environmental signals. One key to understanding the role of tuft cells is that they share many characteristics with the chemosensory cells in taste buds. This suggests that the tuft cells can function as chemoreceptive cells that sense many chemical signals around them. The Dclk1-expressing tuft cell population in the gastric fundus increases in response to oxygenic atrophy, and elevated gastrin levels are necessary for dynamic changes [16].

The population of parietal cells that produce gastric acid and intrinsic factors is the focus of interest. Hydrochloric acid generates an acidic environment (pH < 2) in the gastric lumen. A strongly acidic environment facilitates food digestion, promotes the absorption of minerals, including iron, calcium, and phosphate, and kills food-derived bacteria. High and low levels of gastric acid secretion are potentially injurious to the integrity of the gastric mucosa. Therefore, the gastric mucosa must maintain a balance between gastric acid secretion and mucosal protection. The intrinsic and extrinsic neurohumoral regulation of the stomach balances the effects of the stimulatory (agonist) and inhibitory (antagonist) pathways to maintain a safe range of acid secretion. G cells are gastrin-producing enteroendocrine cells in the antrum that regulate parietal cell function via a feedback pathway from the distal part of the stomach. Gq-coupled receptors (chemical sensors, e.g. CaSR, GPCR6A, and LPAR5) for peptone and amino acids (digested proteins) are present in G cells and are likely to be involved in amino acid-induced gastrin secretion. Gastrin stimulates parietal cells to acid secretion [17–19].

Parietal cells secrete gastric acid via direct and indirect pathways. These cells are directly stimulated by gastrin through activation of the CCK2 receptor [20, 21]. CCK2 receptor is also important for parietal cell differentiation and maturation. Germline CCK2 receptor-deficient mice demonstrate decreased numbers of parietal and ECL cells owing to gastric mucosal atrophy, resulting in increased gastric pH and plasma gastrin levels. However, the primary pathway is the indirect effect of gastrin through the paracrine release of histamine from ECL cells, which directly stimulates parietal cells by activating H2 receptors for acid secretion [6, 22].

Histamine is a biogenic amine generated by histidine decarboxylase in ECL cells present in the corpus of the gastric mucosa. ECL cells are localised next to parietal cells and are closed-type enteroendocrine cells because they have no direct contact with the gastric lumen. Histamine molecules are released directly through these pathways: circulating gastrin stimulates ECL cells via CCK2R, and neuronal pituitary adenylate cyclase-activating peptide stimulates ECL cells via PAC1R. Somatostatin suppresses histamine release via SST2R on ECL cells [23].

**Fig. 2:**
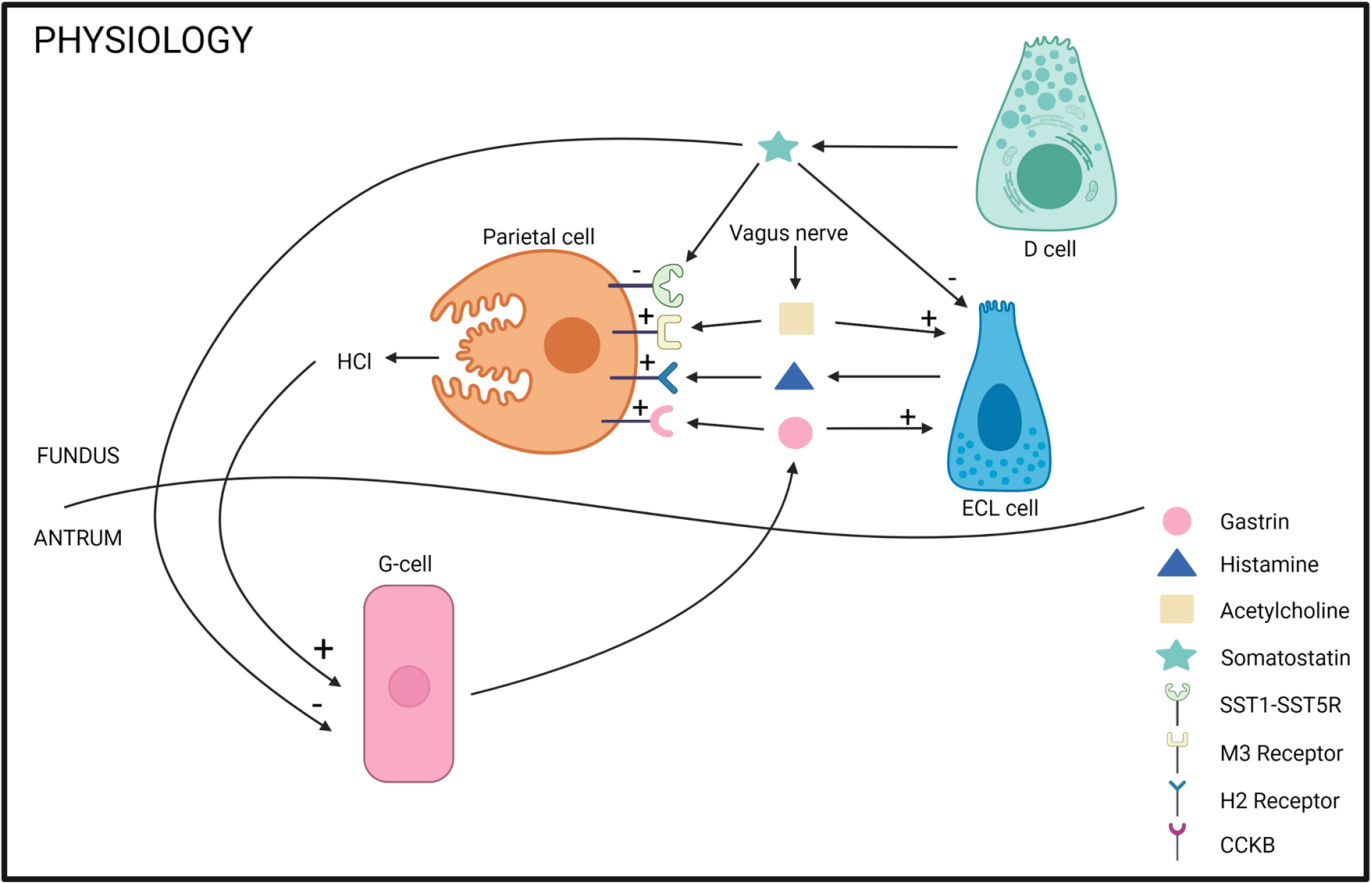
Physiology mechanisms. Pathway parietal cell – G-cell – enterochromaffin-like cell and the regulation. Created with BioRender.com

Gs-coupled histamine H2 receptors are present in parietal cells and directly increase intracellular cAMP levels, stimulating acid secretion. Intraduodenal and intragastric HCl and nutrients stimulate antral D cells to secrete somatostatin, which inhibits gastric acid secretion. Somatostatin receptors (SST1–SST5R) are coupled with inhibitory G proteins and are distributed in the gastric mucosa (e.g. parietal cells, G cells, and ECL cells) (Fig. 2) [24].

**Fig. 3:**
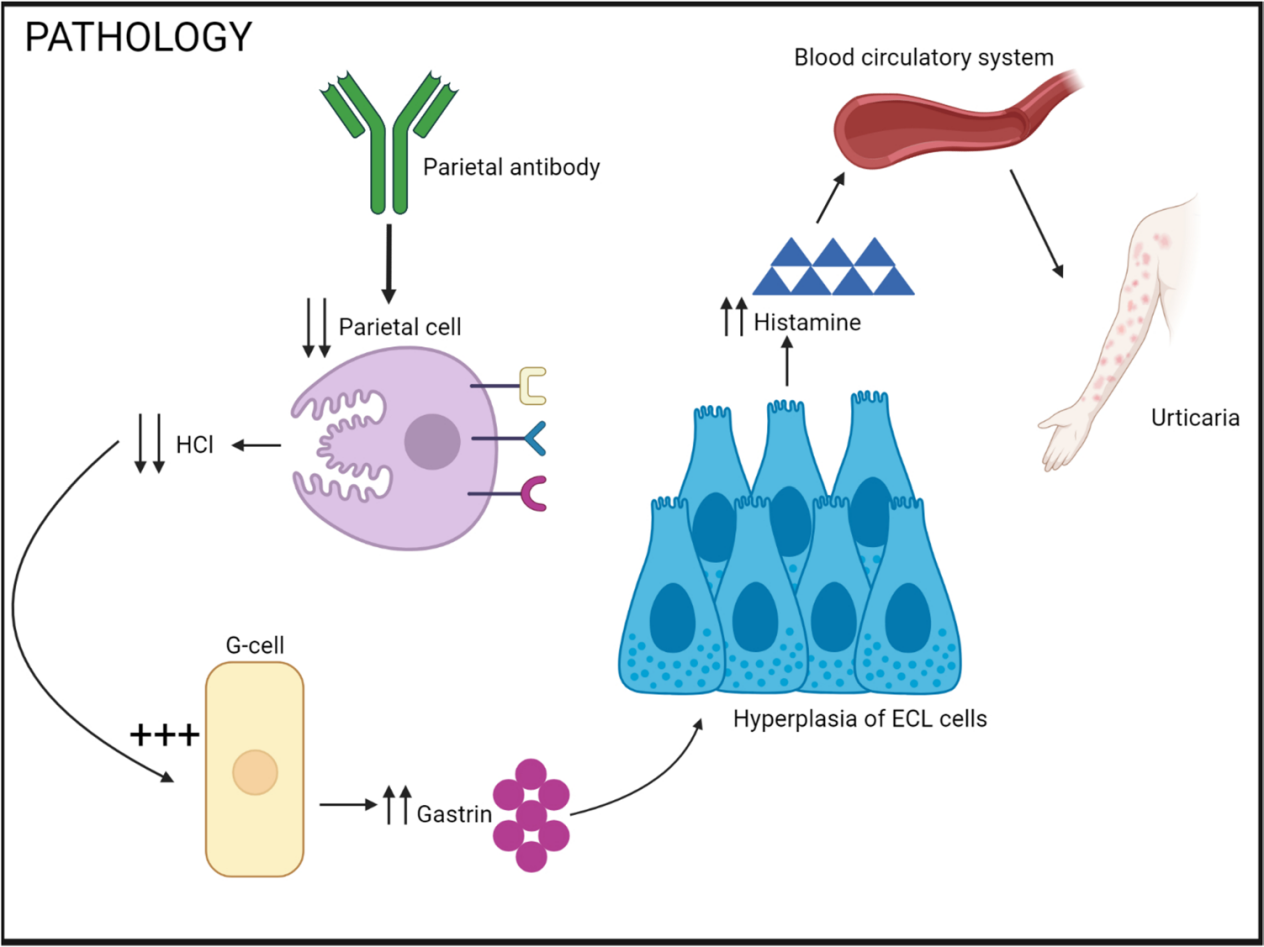
Pathophysiological mechanism of autoimmune gastritis and urticaria. The pathophysiology of chronic spontaneous urticaria in autoimmune atrophic gastritis is complex and involves changes in acid secretion, enterochromaffin-like hyperplasia, ensuing excess of histamine leaks into the bloodstream which could trigger chronic urticaria. Created with BioRender.com

The pathophysiology of CSU in AAG is complex and involves several complex changes in acid secretion, ECL hyperplasia cells, and an excess of histamine that leaks into the bloodstream, which could be a triggering factor in chronic urticaria (Fig. 3). The central point in the pathogenesis of oxyntic mucosal damage is the formation of an autoreactive clone of CD4 + T lymphocytes that stimulates a cellular and humoral immune response against various structures of parietal cells or intrinsic factors. Diffuse destruction of the oxyntic glands results in achlorhydria, pepsinogen deficiency, and a lack of intrinsic factors that participate in the absorption of vitamin B12 [25, 26]. Achlorhydria stimulates G cells in the anthroduodenal region to significantly and long-term increase gastrin secretion. Increased gastrin levels stimulate histamine-producing enhanced ECL cells. Long-term (and in the absence of parietal cells, ineffective) stimulation of ECL cells leads to hyperplasia, possibly even tumour transformation [27]. ECL cell hyperplasia has been reported in children in the AAG group [28]. ECL cell hyperplasia in the stomach may be the cause of CSU because histamine can leak into the bloodstream and cause chronic urticaria. Histamine is a vasoactive mediator and the central factor in the pathophysiology of urticaria. The effects of histamine on cells occur via four receptors: H1, H2, H3, and H4. Histamine receptors are G protein-coupled receptors. The H1 and H2 receptors are at the forefront of interest. The effects of H1 receptors are mediated by increased phospholipase C activity, increased cytoplasmic calcium, and a subsequent increase in protein kinase C activity [29]. H1 receptors are expressed throughout the body, including blood vessels, smooth muscle cells of the airway, and neurons. H1 receptors are activated and lead to allergic/anaphylactic reactions such as vasodilation, pruritus, hypotension, tachycardia, flushing, and bronchoconstriction. They regulate behaviour, memory, learning, locomotion, sleep–wake cycles, thermal regulation, and food intake. H1 receptor antagonists are used to treat the adverse effects of histamine. The effects of H2 receptors are mediated by increased intracellular cAMP levels and the subsequent activation of protein kinase A [30, 31]. H2 receptors are expressed in the parietal cells of the gastric mucosa, smooth muscle cells, and the heart. H2 receptors are activated and primarily lead to increased gastric acid secretion; however, if ECL cells are hyperplastic and parietal cells are damaged in autoimmune gastritis, histamine can leak into the bloodstream.

## Pathological and clinical characterization

**Fig. 4:**
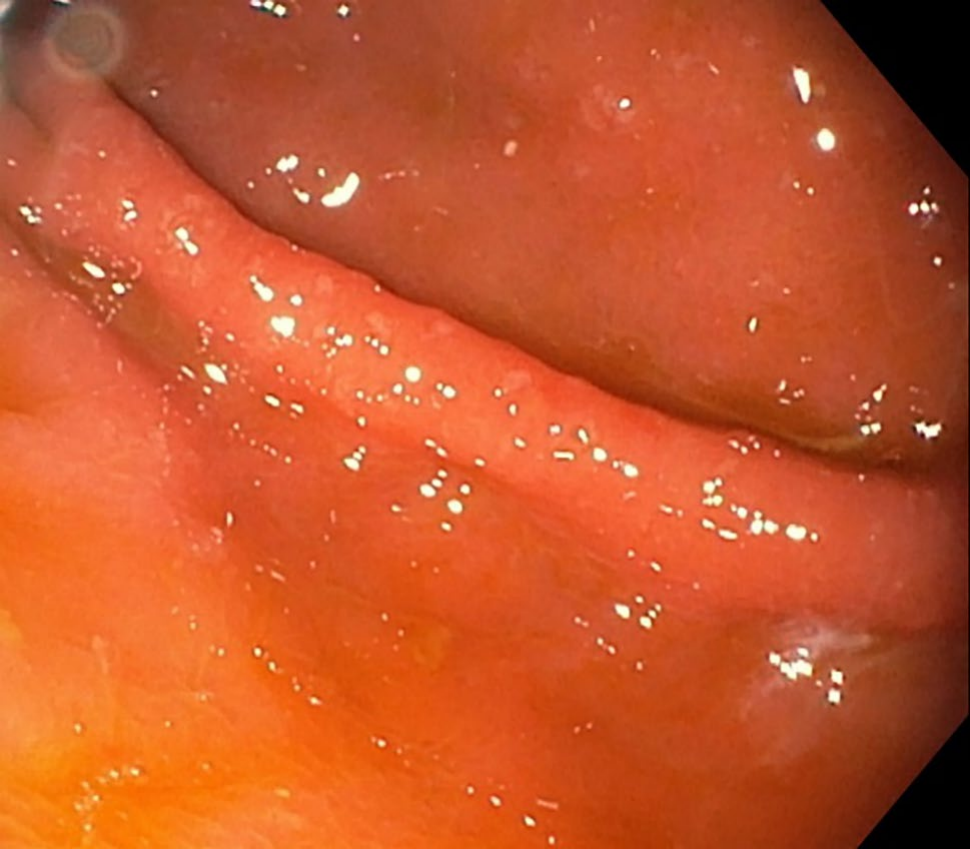
During the endoscopic examination of the stomach, macroscopic diffuse erythema of the body of the stomach with numerous aphthae and atrophy of the antrum was visible. The other findings were normal

**Fig. 5:**
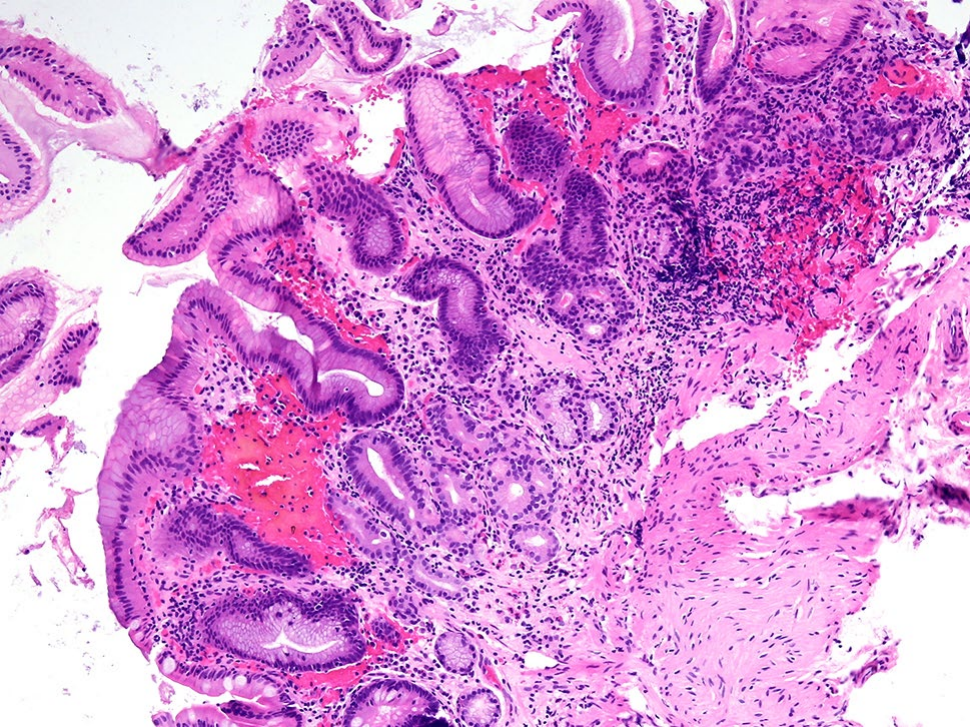
Chronic atrophic gastritis of the gastric body with pseudopyloric and intestinal metaplasia (H&E, 200×)

**Fig. 6:**
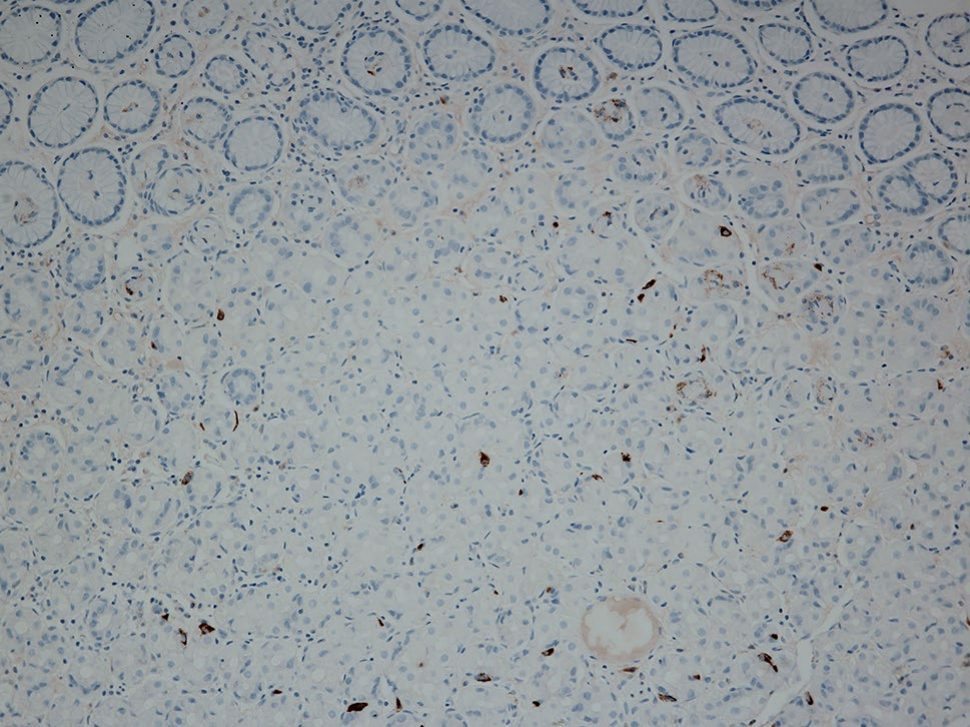
Normal oxyntic gastric mucosa devoid of enterochromaffin-like (ECL) cell hyperplasia. The chromogranin-positive ECL cells are only individually dispersed (chromogranin, 200×)

**Fig. 7:**
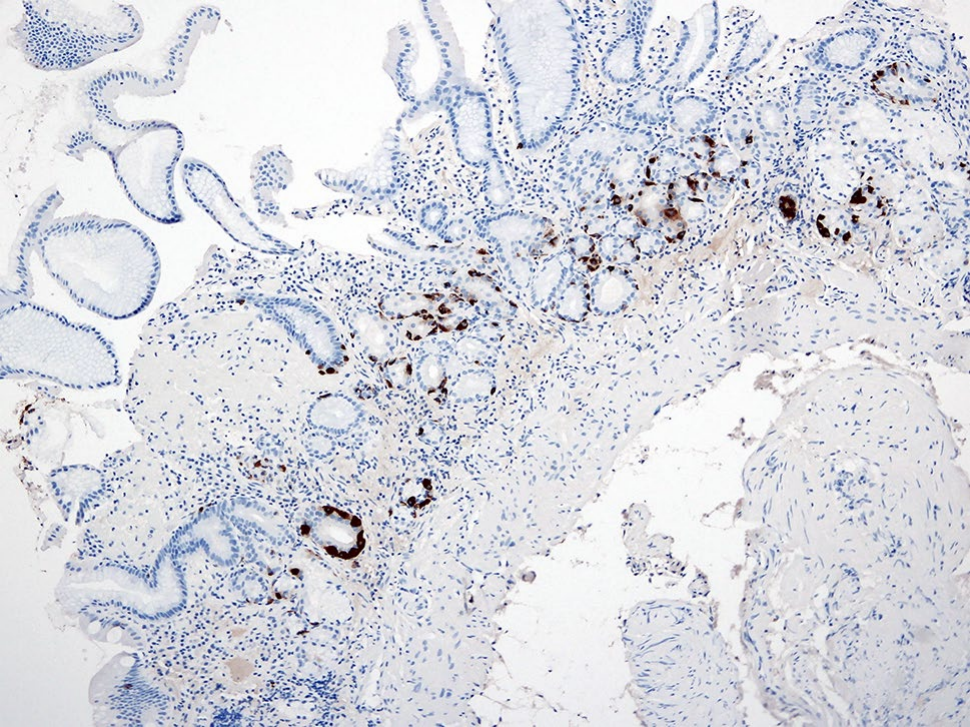
Atrophic mucosa of the gastric body with extensive pseudopyloric metaplasia, focal intestinal metaplasia, and linear hyperplasia of enterochromaffin-like cells (chromogranin, 170×)

Although the actual prevalence of chronic atrophic gastritis is unknown, and this entity may be largely underdiagnosed, patients in whom a diagnosis is established commonly present with advanced disease stages [32]. The diagnosis of AAG is often delayed owing to a lack of specific clinical manifestations and signs and remains reliant on pathological diagnosis. The predominant and characteristic symptoms of AAG include GI symptoms, deficiencies in iron and less commonly vitamin B_12_ [5, 31]. Complications in the clinical course of AAG include ECL hyperplasia, which may precede the formation of a NET from ECL cells due to long-term stimulation by high gastrin levels. Furthermore, adenocarcinoma of the stomach arises from a sequence of changes in intestinal metaplasia–dysplasia–adenocarcinoma. Furthermore, additional risk factors in the pathogenesis of urticaria, including AAG and ECL hyperplasia, need to be identified. A typical endoscopic finding in AAG is diffuse atrophy of the mucous membrane in the body and fundus of the stomach, which is macroscopically visible as the thinning of the mucous membrane with the disappearance of its folds (Fig. 4). Histologically, it presents as inactive atrophic chronic gastritis with pyloric and intestinal metaplasia (Fig. 5). Hyperplasia of neuroendocrine ECL cells indicates hypergastrinaemia in achlorhydria (Figs. 6 and 7). In patients with a histology compatible with autoimmune gastritis, gastroenterologists should consider checking for APCA antibodies and AIFA antibodies to assist with diagnosis. Gastroenterologists should also evaluate anaemia due to vitamin B12 and iron deficiencies.

## Conclusion

Paediatricians and pathologists should consider the possibility of AAG in the paediatric population. This article describes the possible mechanisms underlying urticaria in patients with AAG. These findings suggest that the development of chronic urticaria could be caused by ECL hyperplasia with the subsequent excessive production of histamine. If a paediatric patient presents with symptoms such as anaemia, reduced vitamin B_12_ levels, recurrent urticaria with no other detectable aetiology, positive APCA, and elevated gastrin levels, AAG should be considered a possible cause of these skin manifestations. Physicians should suspect AAG in patients with a history of autoimmune disorders and/or urticaria.
